# Learning Curve and Interobserver Agreement of Confocal Laser Endomicroscopy for Detecting Precancerous or Early-Stage Esophageal Squamous Cancer

**DOI:** 10.1371/journal.pone.0099089

**Published:** 2014-06-04

**Authors:** Jing Liu, Ming Li, Zhen Li, Xiu-Li Zuo, Chang-Qing Li, Yan-Yan Dong, Cheng-Jun Zhou, Yan-Qing Li

**Affiliations:** 1 Department of Gastroenterology, Qilu Hospital, Shandong University, Jinan, China; 2 Department of Anesthesiology, Qilu Hospital, Shandong University, Jinan, China; 3 Department of Pathology, the Second Affiliated Hospital, Shandong University, Jinan, China; University Hospital Llandough, United Kingdom

## Abstract

**Background:**

Confocal laser endomicroscopy (CLE) can provide in vivo subcellular resolution images of esophageal lesions. However, the learning curve in interpreting CLE images of precancerous or early-stage esophageal squamous cancer is unknown. The goal of this study is to evaluate the diagnostic accuracy and inter-observer agreement for differentiating esophageal lesions in CLE images among experienced and inexperienced observers and to assess the learning curve.

**Method:**

After a short training, 8 experienced and 14 inexperienced endoscopists evaluated in sequence 4 sets of high-quality CLE images. Their diagnoses were corrected and discussed after each set. For each image, the diagnostic results, confidence in diagnosis, quality and time to evaluate were recorded.

**Results:**

Overall, diagnostic accuracy was greater for the second, third, fourth set of images as compared with the initial set (odds ratio [OR] 2.01, 95% CI 1.22–3.31; 7.95, 3.74–16.87; and 6.45, 3.14–13.27), respectively, with no difference between the third and fourth sets in accuracy (p = 0.67). Previous experience affected the diagnostic accuracy only in the first set of images (OR 3.70, 1.87–7.29, p<0.001). Inter-observer agreement was higher for experienced than inexperienced endoscopists (0.732 vs. 0.666, p<0.01)

**Conclusion:**

CLE is a promising technology that can be quickly learned after a short training period; previous experience is associated with diagnostic accuracy only at the initial stage of learning.

## Introduction

Esophageal cancer is an important cause of cancer-related deaths worldwide. In 2008, there was an estimated 482,300 new esophageal cancer cases, and 406,800 patients died from the disease worldwide[1]. Although the incidence of esophageal squamous cell carcinoma (ESCC) is decreasing in western countries, the disease is still one of the most prevalent with an incidence of 20.3per100,00 for males,and 8.3per 100,000 for females in Eastern Asia. The prognosis is very poor, with a 5-year survival rate of about 15%[2], and the initial diagnosis of ESCC is often delayed. Early detection of the premalignant state of the disease, such as esophageal squamous intraepithelial neoplasia, and early-stage disease can improve survival [3].

However, standard endoscopy by itself cannot reliably detect squamous dysplasia or early-stage esophageal cancer because of inconspicuous macroscopic appearance of lesions[4]. Therefore, new endoscopic devices are urgently needed for early detection.

Confocal laser endomicroscopy (CLE) is an emerging technique that can provide real-time images of the gastrointestinal epithelium at the subcellular level in vivo[5] [6]. The technique can help detect the disease at an early stage and reduce the biopsy rate,[7] for an instant classification. In addition, treatment is immediate after neoplastic lesions are detected, thus reducing the time and cost for repeat endoscopy[8].

In 2008, Pech et al[9] proposed the cellular and vascular criteria of the early ESCC, then Liu et al[10] described the distinctive features of CLE images for patients with superficial ESCC: an irregular arrangement of squamous epithelial cells, increased diameter of intrapapillary capillary loops (IPCLs), long branching IPCLs and massive IPCLs with tortuous vessels. Recently, Li et al[11] developed a new method – surface maturation score (SMS) -- to distinguish neoplasia from benign areas, which was also proposed to fit the early stage of ESCC.

However, the accuracy of CLE diagnosis depends on the observer's experience[12]. As well, the learning curve of image interpretation must be examined before a new imaging technology is widely used in clinical practice. However, no studies have investigated the learning curve in distinguishing non-neoplastic and neoplastic lesions or whether the endoscopist's experience and ability has an impact on the diagnosis accuracy in squamous intraepithial neoplasia of the esophagus.

We aimed to evaluate the diagnostic accuracy and inter-observer agreement for differentiating esophageal lesions in CLE images among experienced and inexperienced observers and to assess the learning curve. Additionally, we evaluated contributing factors such as image quality, interobserver variability in diagnosis, diagnostic confidence and time needed for diagnosis.

## Materials and Methods

### Endoscopy

From May 2010 to September 2012, 1,345 patients underwent upper gastrointestinal examination by confocal laser endoscopy at Qilu Hospital.

Confocal images were obtained according to our routine procedure. Briefly, 2 experienced confocal laser endoscopists (X.-L.Z. and C.-Q.L.) who had performed more than 500 endoscopies used a confocal laser endomicroscope (EC3870CIK; Pentax, Tokyo, Japan) for endomicroscopy. All procedures were approved by the institutional ethics committee of Qilu Hospital. Written informed consent was obtained from patients before CLE. Before the procedure, patients had fasted for 6 hr and took 80 mg dimethylpolysiloxane orally. Midazolam hydrochloride and meperidine citrate were infused intravenously for sedation. For cases involving esophageal abnormal areas seen by the white-light mode of CLE, 5 ml of 10% fluorescein sodium solution was injected intravenously. CLE images of different depths of suspected lesions were obtained, followed by target biopsy (as described in our previous study[10]). All images were stored in a database as JPEG files without any additional processing with corresponding histopathology results and other information for patients.

### Data collection

A total of 72 patients with 75 lesions had abnormal esophagus lesions seen by the white-light mode of CLE and underwent a biopsy of the squamous epithelium which yielded pathology including normal,hyperplasia,inflammatory,neoplasia,and cancer tissue,but not Barrett's. Good-quality images with no blurring and artifacts were selected from the database by an experienced CLE endoscopist (Z.L.) who had performed more than 300 cases. We selected 2 images with IPCLs for each suspicious site. Finally 72 pairs of images from 69 patients were selected, including 10 of early-stage ESCC, 13 of low-grade neoplasia, 11 of high-grade neoplasia, 31 of inflammation, 2 of normal tissue and 5 of hyperplasia. Images for 3 patients were excluded because they were too dark or too light to be analyzed.

All selected images without patient names and histology and endoscopy results were incorporated into a slideshow (Microsoft Office PowerPoint 2007, Microsoft Inc., USA), and displayed at 19.05×19.05 cm on the screen. Every 2 images represented a suspicious lesion. The slides were displayed to all observers on the same type of computer (Lenovo, Y450, China). Eight of the observers had more than 3 years' experience with CLE, and the remaining 14 observers had more than 3 years' experience in white-light endoscopy but no experience in evaluating CLE images. Neither the experienced nor inexperienced observers participated in the selection of images or had seen the images before the selection.

### Evaluation process

Before the evaluation process, one of the authors (L.M.) gave a half-hour training session consisting of a detailed explanation of the SMS, relevant pathology knowledge of the esophagus, and the image-forming principle of CLE. This training session involved 10 images (5 benign and 5 neoplastic images) that were validated cases used in our previous studies [10]–[11], with corresponding histopathology results. All images used for the training session were not used in the following evaluation process. None of the observers was familiar with other criteria for ESIN or early-stage esophageal cancers.

**Figure 1:**
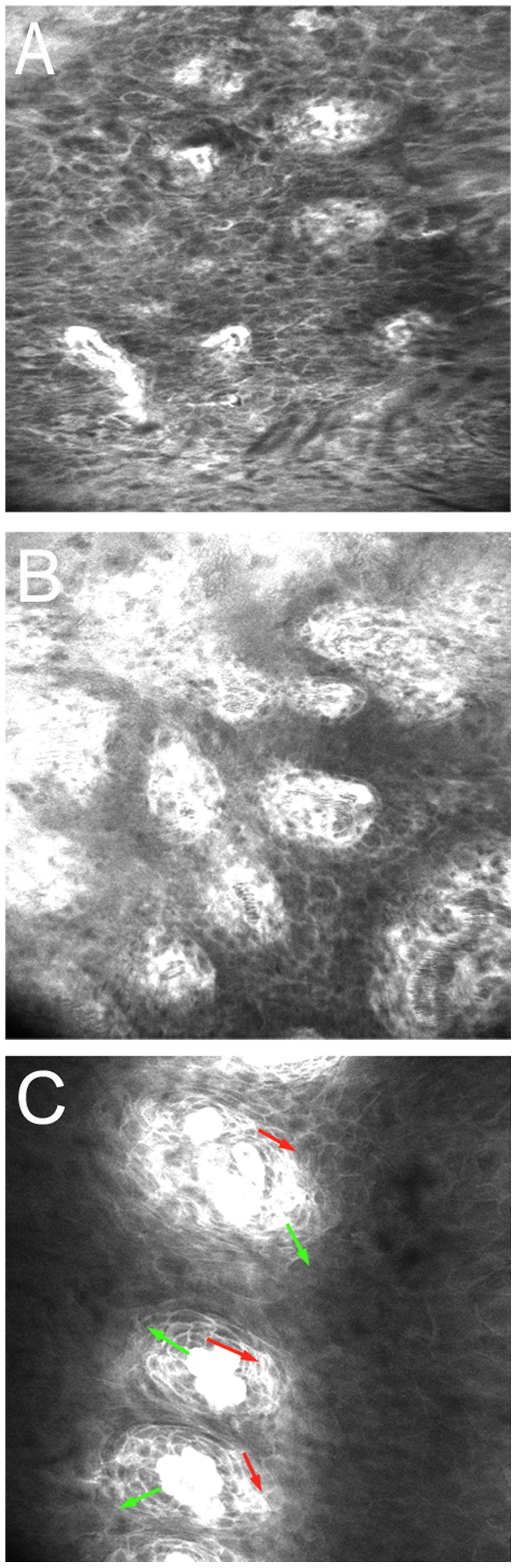
Confocal images of esophageal lesions. A. Confocal image of early-stage esophageal squamous carcinoma without the 4 features of the surface maturation score (SMS). B. Confocal image of low-grade neoplasia (LGN) without the 4 features of the SMS. C. Confocal image of reflux esophagitis, with the 2 SMS features existence and gradient but not polarity or compass effect, the red arrows show existence, and the green arrows show gradient.

All 72 pairs of images were randomly divided into 4 sets (n = 18 pairs each) by use of computer-generated sequence numbers. For each pair of images, observers who were blinded to patient characteristics, history, and other data independently commented on the presence or absence of the 4 SMS features for diagnosis; discussions were not permitted during the evaluation process. Finally, all observers gave an overall diagnosis according to the SMS. The SMS involves 4 features: existence, gradient, polarity and compass effect. In early-stage ESCC or ESIN (Figure 1A, 1B), the 4 features are absent, whereas in benign lesions, at least 1 feature is present (Figure 1C). The CLE images were defined as “neoplasia,” including high-grade dysplasia, low-grade dysplasia, and early-stage ESCC, if the 4 major features were absent (SMS = 0).

The confidence level was recorded as 1, guess; 2, moderately sure; and 3, positively sure. The evaluation time was defined as from when the images were shown to the observers to when the diagnosis was completed and was recorded in a Microsoft Excel table (Microsoft Office Excel 2007, Microsoft Inc, USA).

Pairs of images were scored for overall quality as 3, excellent (IPCL and cell visualization sure and clear); 2, good (IPCL and cell visualization sure but unclear); or 1, moderate (IPCL and cell visualization unsure and unclear).

After scoring each set of 18 pairs of images, the histopathology results were disclosed to the observers, and every image was discussed, especially the incorrectly diagnosed ones. Then every observer took a 15-min rest before the next evaluation process.

### Reference standard

All targeted biopsy specimens were assessed by an experienced gastrointestinal pathologist (C.-J.Z) who was blinded to the history of patients, the CLE images and endoscopy results, according to the modified Vienna classification of gastrointestinal epithelial neoplasia[13]. We used the histological diagnosis of all biopsies as the reference standard diagnosis.

### Statistical analysis

The accuracy, sensitivity and specificity for interpreting CLE images were calculated according to the STARD statements for diagnostic accuracy studies[14]. Two-tailed p<0.05 was considered statistically significant. The differences in diagnostic accuracy and diagnostic time between inexperienced and experienced observers were compared by chi-square analysis and one-way ANOVA respectively.

The learning curve of inexperienced observers was established by logistic regression analysis of the association of image-set sequence and diagnostic accuracy. Multilevel logistic regression analysis, with the 1st level representing each diagnosis and the 2nd level representing each observer, was used to examine the effect of observer experience level (0 for inexperienced, 1 for experienced), observer confidence, image quality, and training-set order (1 to 4) on diagnostic accuracy. Multiple logistic analyses involved use of MLwin 2.26 (University of Bristol, Bristol, England). The figures in this article were created with GraphPad Primer 5.0.

To evaluate the level of agreement, the multirater k statistic was calculated by use of the SPSS mkappasc.sps macro (available at http://support.spss.com). The strength of agreement was defined as follows[15]: slight (κ<0.2), fair (κ 0.201–0.4), moderate (κ0.401–0.6), substantial (κ 0.601–0.8) and excellent (κ 0.801–1.0). Statistical analysis involved use of SPSS 16.0 for Windows (SPSS Inc., Chicago, IL). P<0.05 was considered statistically significant.

## Results

### The accuracy of observers

**Figure 2:**
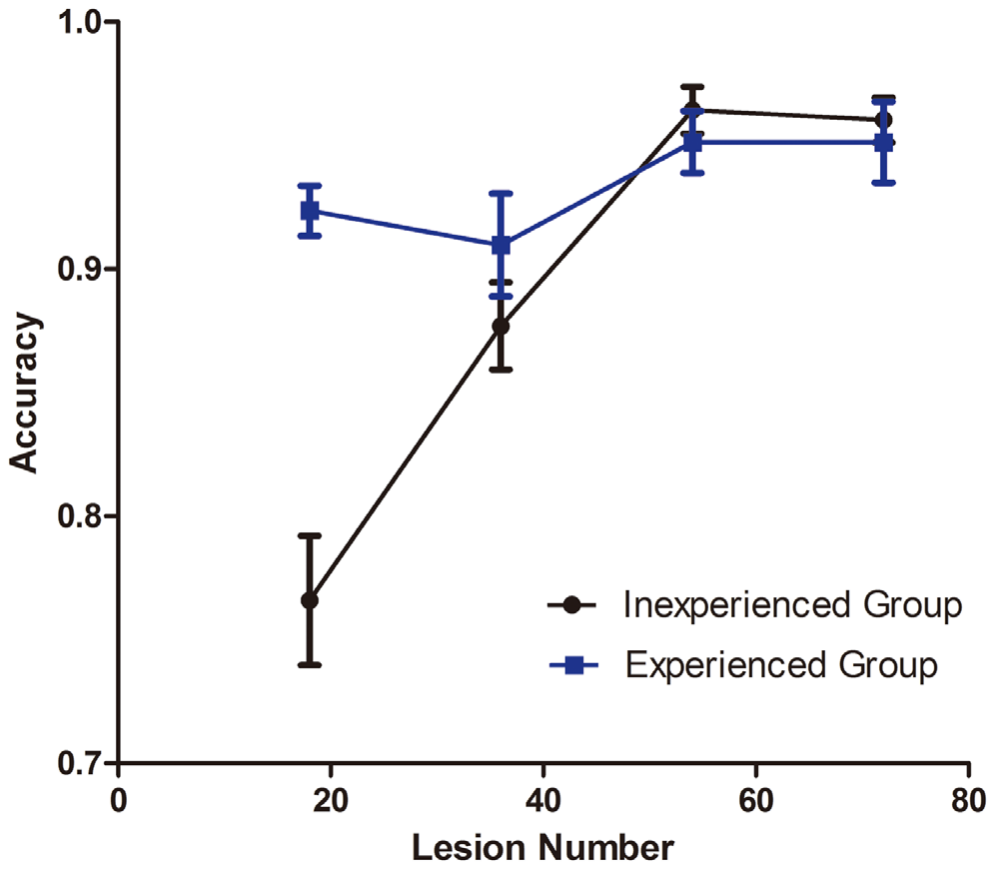
Diagnostic accuracy for inexperienced and experienced endoscopists for 4 sets of confocal laser endoscopy (CLE) images. Data are mean±SD.

**Figure 3:**
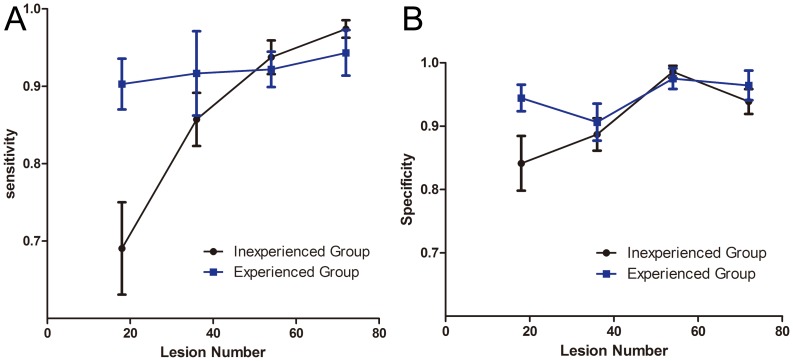
Diagnostic sensitivity and specificity for two groups of endoscopist. A. Diagnostic sensitivity for inexperienced and experienced endoscopists for 4 sets of CLE images. B. Diagnostic specificity for inexperienced and experienced endoscopists for 4 sets of CLE images. Data are mean±SD.

The overall accuracy for interpreting dysplasia in esophagus lesions was 90.7% 95% confidence interval [95% CI] 88.9%–92.6%), with significant differences for experienced observers (93.4%, 91.7%–95.0%) and inexperienced observers (89.2%, 86.4%–91.9%, p<0.05) (Table 1,Figure 2). The accuracy was higher for only the first set of images for experienced than inexperienced observers (92.4%, 90.0%–94.8%, vs 76.6%, 70.9%–82.2%, p<0.05).The sensitivity, specificity for expert and non-expert in different sets of images were calculated and compared as shown in Table 1,Figure 3A,Figure 3B.

**Table 1:** Sensitivity,specificity,accuracy for experienced and inexperienced observers.

		Sensitivity%(95%CI)	Specificity%(95%CI)	Accuracy%(95%CI)
The 1 set	experienced	90.3 (82.5∼98.0)	94.4 (89.5∼99.4)	92.4 (90.0∼94.8)
	in-experienced	69.0 (56.2∼81.9)	84.1 (74.8∼93.4)	76.6(70.9∼82.2)
	p	0.19	0.1	<0.01
The 2set	experienced	91.6 (78.7∼105.0)	90.6 (83.7∼97.5)	91.0 (86.0∼95.9)
	in-experienced	85.7 (78.3∼93.1)	88.7 (83.2∼94.2)	88.7 (83.9∼91.5)
	p	0.342	0.638	0.259
The 3 set	experienced	92.2 (82.8∼97.2)	97.5 (93.6∼101.4)	95.1 (92.2∼98.1)
	in-experienced	93.8 (89.1∼98.4)	98.6 (96.5∼100.7)	96.4 (94.4∼98.5)
	p	0.647	0.553	0.420
The 4 set	experienced	94.3 (88.6∼99.9)	96.4 (90.9∼102.0)	95.1 (92.2∼98.1)
	in-experienced	97.4 (94.9∼99.9)	93.9 (89.6∼98.1)	96.0 (94.1∼98.0)
	p	0.260	0.427	0.609
The total	experienced	92.1 (88.6∼95.6)	94.7 (92.3∼97.1)	93.4 (91.8∼95.0)
	in-experienced	86.5 (81.9∼91.1)	91.3 (88.3∼94.3)	89.2(86.4∼91.9)
	p	0.093	0.123	0.029

### Effect of previous experience on the interpretation of esophageal lesions

As compared with no experience, previous experience was associated with diagnostic accuracy in detecting dysplasia of esophageal lesions only for the first set of images (odds ratio [OR] 3.70, 95% CI 1.87–7.29), p<0.001). Overall, across the whole evaluation process, previous experience was associated with diagnostic accuracy for ESIN or early-stage ESCC (OR 1.77, 1.20–2.60, p = 0.002) (Table 2).

**Table 2:** Association of experience with the diagnostic accuracy in the four set of images.

	Set1 OR95%CI	Set2 OR95%CI	Set3 OR95%CI	Set4 OR95%CI	Total OR95%CI
In-experiencedobservers	1.00	1.00	1.00	1.00	1.00
Experienced observers	3.70 95%CI 1.87∼7.29	1.54 95% CI 0.77-3.11	0.72 95%CI 0.26–1.99	0.81 95% CI 0.30-2.17	1.77 95%CI 1.20-2.60
p	<0.001	0.11	0.26	0.34	0.002

### Assessment of learning curve

**Figure 4:**
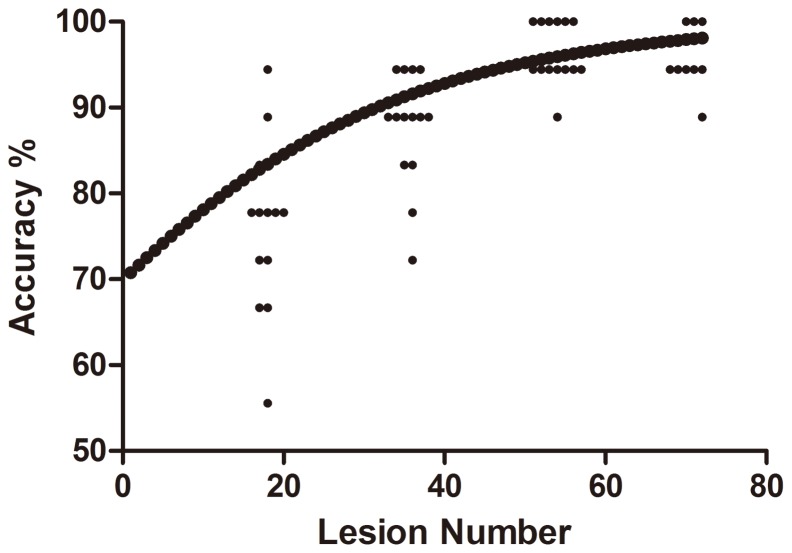
Logistic regression curve of accuracy in detecting number of precancerous or early-stage esophageal squamous cancer lesions by inexperienced endoscopists. Individual points are the accuracies for each inexperienced observer for each set of CLE images (1–18, 19–36, 37–54, 55–72).

For inexperienced observers, diagnostic accuracy was better for the second, third, and fourth than first set of images (OR 2.01, 95% CI 1.22–3.31, p<0.01; 7.95, 3.74–16.87, p<0.01; 6.45, 3.14–13.27, p<0.01, respectively), with diagnostic accuracy better for only the third than second set of images (OR 7.95, 3.74–6.87 vs. 2.01, 1.22–3.31, p<0.01) (Table 3). A logistic regression curve for inexperienced observers is in Figure 4.

**Table 3:** Association of set sequence, confidence level,and image quality with diagnostic accuracy.

	Experienced observer (0R) 95% CI	Inexperienced observers(0R) 95% CI	Total (0R) 95% CI
Set1	1.00	1.00	1.00
Set2	0.82(0.32–2.14)	2.01(1.22–3.31)	1.63 (1.05–2.51)
Set3	1.60(0.55–4.62)	7.95(3.75–16.87)	4.84 (2.70–8.68)
Set4	1.68(0.57–4.92)	6.45(3.14–13.27)	4.28(2.41–7.58)
Confidence1	1.00	1.00	1.00
Confidence2	1.81(0.72–4.51)	1.66(0.93–2.97)	1.7(1.06–2.75)
Confidence3	14.76(3.82–56.97)	4.26(2.15–8.45)	5.68(3.20–10.09)
Quality1	1.00	1.00	1.00
Quality 2	2.23(0.95–5.25)	1.59(0.93–2.70)	1.69(1.09–2.62)
Quality 3	5.46(1.29–23.16)	2.39(1.27–4.49)	2.54(1.48–4.35)

For experienced observers, diagnostic accuracy was better for the third and fourth fourth sets of images (OR 1.60, 95% CI 0.55–4.62, p = 0.19; 1.68, 0.57–4.92, p = 0.17, respectively).

### Accuracy and confidence

From the multiple logistic regression model, the confidence level of observers was strongly associated with diagnostic accuracy (OR 5.68, 95% CI 3.20–10.09 p<0.001), particularly with experienced observers when they were positively sure about their decisions (OR 14.76, 95% CI 3.83–56.97) (Table 3). If the experienced observers were positively sure of their diagnosis, the accuracy was high (98.8%, 97.1–99.6%). For inexperienced observers, confidence was associated with diagnostic accuracy when observers were positively sure about their decisions (OR 4.26, 2.15–8.45) but less so than for experienced observers. The accuracy could reach 94.7% (92.5–96.3%) when the inexperienced observers were positively sure of their interpretation.

### Impact of image quality on accuracy

Image quality was an important predictor of correct diagnosis of esophageal lesions (OR 2.54, 95% CI 1.48–4.35) (Table 3). As compared with all images, for images with excellent quality, accuracy was higher for both experienced and inexperienced observers (99.04 vs. 93.4%, p<0.001; 93.1 vs. 89.2%, p<0.05). Further, the association was more pronounced for experienced than inexperienced observers (OR 5.46, 95% CI 1.29–23.16) vs. 2.39, 1.27–4.49).

### Interobsever agreement

For inexperienced and experienced observers, the overall interobserver agreement was substantial (k = 0.666, 95% CI 0.642–0.690; k 0.732, 0.688–0.776) but differed significantly (p<0.01) (Table 4). With the learning process, the k value for inexperienced observers increased from fair (k 0.347, 95% CI 0.298–0.395) to almost excellent (0.850, 0.801–0.898) from group 1 to 3 images and remained stable for group 4 images (0.856, 0.807–0.904) (Table 4). The k value for experienced observers was significantly higher only for the first set of images (p<0.01), but the learning process abolished the difference for later sets.

**Table 4:** The interobserver agreement in different sets of images.

	Set 1 k	Set 2 k	Set 3 k	Set 4 k	Total k
Experienced group	0.718 (0.631∼0.806)	0.536 (0.449∼0.623)	0.8318 (0.745∼0.919)	0.840 (0.752∼0.927)	0.732 (0.688∼0.776)
Inexperienced group	0.347 (0.298∼0.395)	0.554 (0.506∼0.602)	0.850 (0.801∼0.898)	0.856 (0.807∼0.904)	0.666 (0.642∼0.690)
p	<0.01	0.116	0.120	0.148	<0.01

### Diagnosis time

**Figure 5:**
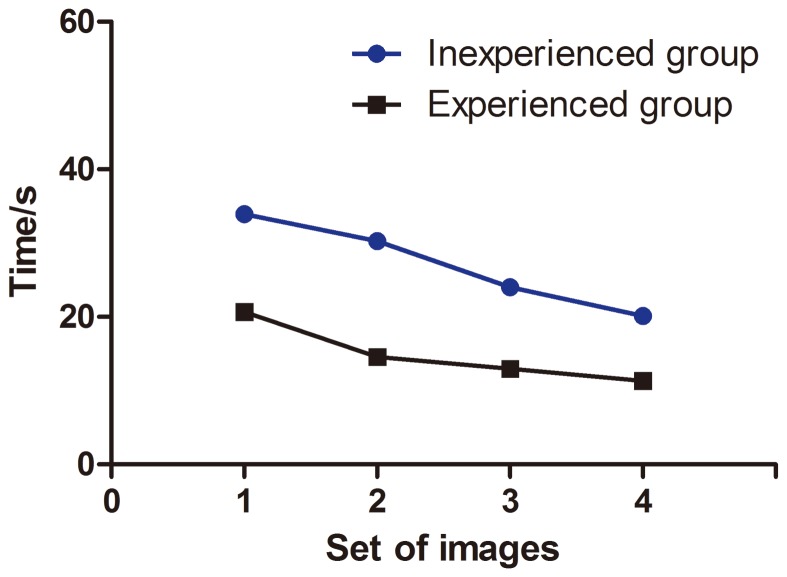
Time to interpret pairs of CLE images for inexperienced and experienced endoscopists for 4 sets of images. Data are mean±SD.

The mean time for interpreting each pair of images was higher for inexperienced than experienced observers (27.07 sec, 95% CI 26.01–28.13 sec vs. 14.82 sec, 13.96–15.67 sec, p<0.01). The time spent on later sets decreased for the inexperienced observers (p<0.001) (Figure 5).

## Discussion

CLE can allow for reliable *in vivo* classification during endoscopy. However, its application for classification requires an endoscopist's expertise in image reading. Thus, we aimed to evaluate the learning curve for interpreting CLE images of ESIN and early-stage ESCC and how experience affects the diagnostic accuracy. Among our 22 observers of a large sample of images, the correctclassification of CLE images could be learned quickly after a short-term training and learning process.

Gaddam et al[16] found a short learning curve for detection of Barrett's esophagus in CLE images. Kuiper et al[17] demonstrated that differentiating colorectal lesions in CLE images can be learned quickly with a detailed description of the Mainz classification and a set of 10 images. The learning curve with CLE images for predicting colorectal neoplasia was evaluated among a wide range of gastrointestinal specialists [18].

Lim et al[12] demonstrated that experience had an impact on accuracy in the diagnosis of gastric intestinal metaplasia(GIM)and gastric carcinoma using CLE. By evaluating the association of previous experience in CLE image interpretation and diagnostic accuracy, we also found that previous experience affected accuracy in classification of ESIN or early-stage ESCC in the initial stage of image interpretation. However, even with a short training period and 18 pairs of images, the effect of previous experience on diagnostic accuracy was not significant.

Our study confirmed that SMS method was a criterion with high accuracy and substantial interobserver agreement in classification of esophageal lesions and it can be learned quickly after a short training. We also demonstrated that the SMS method is appropriate for detecting early-stage ESCC with a high sensitivity (91.3% and 88.6% for experienced and inexperienced endoscopists, respectively). We did not choose other criteria of CLE to evaluate the learning curve for interpreting ESIN or ESCC because the SMS method cannot be used to judge diameter or morphology of IPCLs and can be used more easily in the clinic than can other criteria. The SMS method is suitable for early-stage ESCC and ESIN.

The interobserver agreement was higher among experienced than inexperienced observers for the first set but not subsequent sets of images. Thus, interobserver agreement may improve after a short learning process. Unlike previous study[12]–[16], our data showed that interobserver agreement was lower but not significantly for experienced than inexperienced observers for the last 3 sets of images. The number of observers was lower in the experienced than inexperienced group, so the data may not be stable.

Similar to previous studies[16]–[17], we examined confidence in the image reading process. Accuracy could be higher when observers were very confident of their classification (experienced 98.8%, inexperienced 94.7%). Thus, use of CLE may reduce unnecessary biopsies when observers are confident of their classification. Also, when the image quality was excellent, both experienced and inexperienced observers showed high diagnostic accuracy. High-quality images may be associated with high diagnostic accuracy, and thus increasing the image quality may increase the efficiency of CLE in clinical practice.

The accuracy of our study was higher than in a previous study[11] perhaps because we excluded low-quality images. We excluded data for 3 patients because the quality of images was too low for interpretation by the experienced endoscopist, which may influence the learning session.

In this study, we found that experienced endoscopists are affected more seriously by the image quality. The reason might lie in learning effect. When assessing low-quality image, the experience of learning was reduced by artifacts and poor contrast. However, for high-quality image, the learning effect would be introduced to increase the diagnostic accuracy, which explains the differences between experienced and inexperienced.

Our study contains several limitations. Unlike previous study, our research did not use the test-retest procedure.The wide use of CLE involves interpretation of images and also CLE performance. Many studies have investigated the procedure of ultrasonic gastroscopy and laparoscopy[19], but study of the learning curve of CLE is limited, and further study should be done. We used a post-procedure analysis rather than real-time evaluation, and the endoscopists were blinded to other information such as age of patients and results of white-light endoscopy, so the results do not reflect the reality of clinical practice. Accuracy of real-time assessment may be higher[20]–[21]or lower[22]–[23] than with post-procedure assessment. Low-quality images are usually obtained in clinical practice, but in our study, all images were selected by an experienced CLE endoscopist and had relative high quality, which may improve the diagnosis accuracy and cannot reflect the condition in clinical use. However, because our study was of the learning curve, inexperienced observers should first evaluate high-quality images that are representative of lesions[17]. Further study with all kinds of images or real-time research should be done. Another limitation of our study is that we did not distinguish low- and high-grade neoplasia and early-stage ESCC. No previous study has made such distinction. Further study should evaluate the distinction between low- and high-grade neoplasia. We believe that a fluorescein-based system does not allow for differentiating cytonuclei features of the epithelium. Acriflavine is a good agent to show cytonuclei features but is considered a potential carcinogenic agent; further study should evaluate a new cytonuclei staining agent for CLE to distinguish low- and high-grade neoplasia. Furthermore, these parameters were established by eCLE and additional studies for validation may be required for the pCLE setting.

In conclusion, this study confirmed that CLE images of ESIN and early-stage ESCC can be interpreted after a short training and learning curve, and previous experience influences diagnostic accuracy at the initial stage but not later stages of the learning process. The learning curve for performing the CLE procedure and real-time interpretation of images should be evaluated in further study.
